# Coronary stenosis assessment: AI-based CT quantification, visual analysis of invasive angiography, and quantitative coronary angiography

**DOI:** 10.1186/s13244-026-02308-2

**Published:** 2026-05-16

**Authors:** Lihua Yu, Mingyuan Yuan, Xu Dai, Yarong Yu, Jiajun Yuan, Chao Zheng, Lei Xu, Ting Liu, Jiayin Zhang

**Affiliations:** 1 Department of Radiology, Shanghai General Hospital, Shanghai Jiao Tong University School of Medicine, Shanghai, China; 2 Faculty of Medical Imaging Technology, College of Health Science and Technology, Shanghai Jiao Tong University School of Medicine, Shanghai, China; 3 Department of Radiology, Affiliated Zhoupu Hospital, Shanghai University of Medicine and Health Science, Shanghai, China; 4 Shukun Technology Co., Ltd, Beijing, China; 5 Department of Radiology, Beijing Anzhen Hospital, Beijing Institute of Heart, Lung, and Blood Vessel Diseases, Capital Medical University, Beijing, China; 6 Department of Radiology, The First Hospital of China Medical University, Shenyang, China

**Keywords:** Coronary artery disease, Coronary computed tomography angiography, Artificial intelligence, Invasive coronary angiography, Quantitative coronary angiography

## Abstract

**Objective:**

To evaluate the diagnostic performance for coronary stenosis of artificial intelligence (AI)-based CT quantification, manual CT quantification, and visual invasive coronary angiography (ICA) assessment against quantitative coronary angiography (QCA).

**Materials and methods:**

This retrospective study included patients who underwent both coronary CT angiography (CCTA) and ICA within 1 month. Diameter stenosis (DS) was quantified on CCTA images both automatically by a deep-learning model and manually by radiologists, and was assessed visually on ICA. Diagnostic performance for detecting obstructive stenosis (DS ≥ 50% and ≥ 70%) was assessed against QCA using receiver operating characteristic (ROC) curve analysis on per-patient, per-vessel, and per-segment levels. Agreement with QCA was evaluated using Bland-Altman analysis.

**Results:**

A total of 368 patients were included. AI-based CT quantification demonstrated high diagnostic accuracy. For DS ≥ 50%, area under the curves (AUCs) were 0.93 (per-patient), 0.94 (per-vessel), and 0.96 (per-segment). For DS ≥ 70%, AUCs were 0.85, 0.89, and 0.90, respectively. AI-based CT quantification significantly outperformed manual CT and visual ICA assessments at most levels in terms of AUC (all *p* < 0.05), except vs manual quantification by a senior radiologist at the segment level for ≥ 70% stenosis (*p* = 0.060). Bland-Altman analysis showed superior agreement between AI-based CT quantification of DS and QCA (mean differences: 1.5% per-patient, −0.7% per-vessel, −1.2% per-segment) compared with manual CT quantification and visual assessment of ICA.

**Conclusions:**

AI-based CT quantification demonstrated high diagnostic performance for obstructive stenosis with good agreement against QCA, outperforming manual CT quantification and visual assessment of ICA in most scenarios, with the exception of segment-level assessment of ≥ 70% DS compared with manual quantification by senior radiologists.

**Critical relevance statement:**

AI-aided CT quantification outperforms both manual CT quantification and visual ICA analysis in diagnosing obstructive stenosis using QCA as the reference standard, providing an objective tool to reduce diagnostic uncertainty and guide subsequent patient management.

**Key Points:**

- Manual coronary stenosis quantification is subject to significant inter-observer variability.
- AI-based CT quantification outperforms both manual CT quantification and visual ICA analysis.
- Discordance between AI results and the angiographer’s visual estimation should prompt a physiological evaluation.

**Graphical Abstract:**

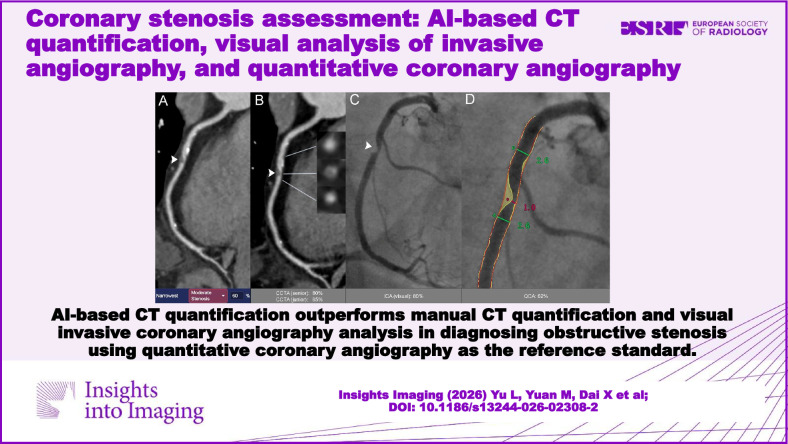

## Introduction

Ischemic heart disease, primarily attributed to obstructive coronary artery disease (CAD), stands as a primary contributor to global mortality [1]. Percutaneous coronary intervention (PCI) is the main therapeutic approach for obstructive CAD [2, 3]. The decision to perform PCI is critically contingent upon precise evaluation of stenosis severity, underscoring the importance of accurate coronary stenosis assessment in clinical management [2, 4].

Coronary CT angiography (CCTA), a commonly employed noninvasive imaging modality, has been shown to reliably rule out obstructive CAD, with current guidelines endorsing it as the first-tier method for evaluation of CAD [5, 6]. While CCTA demonstrates high sensitivity and negative predictive value (NPV) for obstructive CAD, its diagnostic specificity and positive predictive value (PPV) remain only moderate [6]. These limitations are largely attributable to technical factors, such as restricted spatial resolution and blooming artifacts caused by coronary calcifications [7–9]. Thus, the role of CCTA in determining invasive treatment strategy is limited.

Invasive coronary angiography (ICA) is considered the “gold standard” for the diagnosis of coronary stenosis. Currently, visual evaluation of the degree of stenosis determined by the ICA is the standard method for guiding revascularization, but ad hoc visual assessment of angiograms has high variability [2, 4]. Quantitative coronary angiography (QCA), which is a technique providing analysis of angiograms, provides more objective and reproducible measurements of stenosis [10]. Nevertheless, despite these benefits, the widespread clinical use of QCA is limited by its time-consuming workflow, dependence on high-quality angiographic images, and the need for specialized software and technical expertise [10].

Recently, artificial intelligence (AI)-aided models have played increasingly important roles in automatic segmentation of coronary arteries and detection of stenosis. By leveraging deep learning (DL) algorithms and large annotated imaging datasets, AI-based models can extract subtle imaging features beyond the perception of human readers. Specifically, they are capable of identifying fine gray-scale gradients and contextual patterns for robust vessel segmentation and delivering pixel-level accuracy in lumen quantification, which is often subject to human variability. Such advanced capabilities reduce observer variability and provide highly reproducible measurements, thereby enhancing the accuracy and consistency of image interpretation [11–13]. Given these advantages, previous studies have revealed that AI-based CCTA may enable precise stenosis assessment and plaque quantification against an invasive reference standard [14, 15].

Given the aforementioned evidence, we hypothesized that an AI-aided CCTA strategy may have the potential to offer more accurate and reproducible stenosis quantification than does ICA, therefore guiding optimal treatment strategy. The aim of this study was to evaluate the diagnostic performance of stenosis quantification by AI-aided CCTA and visual analysis of ICA, with reference to QCA.

## Materials and methods

### Study population

The institutional review board of the Shanghai General Hospital approved this retrospective study, and written informed consent from patients was waived (reference number 2023-049). We retrospectively screened the database from four tertiary hospitals between January 2019 and August 2022. CCTA was clinically indicated in symptomatic patients with intermediate pre-test probability of CAD or known CAD. The inclusion criterion was that patients with suspected or known CAD who underwent CCTA and ICA within 1 month. The exclusion criteria were (1) patients with a previous history of coronary bypass grafting or stenting; (2) severely impaired image quality of CCTA, defined as non-diagnostic examinations. The image quality was assessed using a 4-point Likert scale: 4 = excellent (absence of artifact), 3 = good (presence of mild artifact), 2 = sufficient (presence of moderate artifact, but still diagnostic), 1 = poor (presence of severe artifact, non-diagnostic).

### CCTA protocol

CCTA was performed on five CT scanners with 64-detector rows or greater from three vendors**:** (1) third-generation dual-source CT (SOMATOM Force, Siemens Healthineers); (2) second-generation dual-source CT (SOMATOM Definition Flash, Siemens Healthineers); (3) first-generation dual-source CT (SOMATOM Definition, Siemens Healthineers); (4) 256-row wide detector CT scanner (Revolution CT, GE Healthcare); and (5) 320-row wide detector CT scanner (Aquilion ONE, Toshiba). The details of imaging parameters are given in Supplementary Files—extended methods. Heart rate control was achieved with the administration of beta-blockers when the heart rate exceeded 80 beats per minute, unless contraindicated. Sublingual nitroglycerin was not routinely administered prior to image acquisition. Patients with significant arrhythmia that precluded reliable image acquisition were excluded, whereas mild or occasional rhythm irregularities were permitted if overall image quality was considered diagnostic. The image phase with optimal image quality was subsequently selected and used for further analysis.

### Manual CT quantification

For manual quantification, the dataset was first transferred to the dedicated commercially available workstation (Syngo.Via, version VB20, Siemens Healthineers) for core lab reading. Coronary arteries with a diameter ≥ 2 mm were evaluated according to the 18-segment model of the Society of Cardiovascular Computed Tomography (SCCT) [16]. The diameter stenosis (DS) was defined as (reference diameter - minimal lumen diameter)/reference diameter. The proximal and distal vessel diameter was measured manually on cross-sectional images, immediately proximal/distal to the lesion, where no plaque could be detected. The reference diameter was determined as an average of the proximal and distal vessel diameters. The minimal lumen diameter was measured manually at the narrowest level of the lesion on the cross-sectional images. The stenosis severity was also recorded as categorical variables of normal (0%), minimal (1%–24%), mild (25%–49%), moderate (50%–69%), severe (70%–99%), or occluded (100%). For per-patient, per-vessel, and per-segment analyses, where there were multiple lesions, the lesion with maximal DS within a patient, vessel, or segment was used.

Two senior cardiovascular radiologists (with 14 and 10 years of experience in cardiovascular imaging, having each interpreted approximately 10,000–12,000 CCTA examinations) and two junior cardiovascular radiologists (with 6 and 5 years of experience in cardiovascular imaging, having each interpreted approximately 3000–3500 CCTA examinations) independently evaluated all segments without knowing the clinical history and the results of ICA and automated CCTA quantification. The mean DS value obtained from two senior cardiovascular radiologists was designated as CCTA (senior), while the mean DS value obtained from two junior cardiovascular radiologists was designated as CCTA (junior).

### AI-aided model for automated CCTA quantification

A commercially available AI system (CoronaryDoc, V5.1.2, Shukun Technology) was utilized to automate the entire coronary imaging workflow, encompassing segmentation, centerline tracking, branch identification, vessel reconstruction, plaque detection, and stenosis assessment. Customized 3D V-net architectures were employed for segmenting cardiac chambers and coronary arteries, enabling the automated classification of three major coronary branches and 18 segments. A fine-tuned 16-layer VGG model was applied to detect atherosclerotic plaques using data from straightened vessels, while a 2D U-net facilitated lumen segmentation of the coronary artery. The segmented lumen data were used to generate mean diameter curves along the artery centerline. Curved planar reformation (CPR) was utilized to visualize the tortuous vessels in 2D, and a fast ray-casting technique produced volume-rendered (VR) images, yielding accurate 3D representations with spatial coherence and enhanced color differentiation [17]. Details of the AI system are provided in the Supplementary Files—extended methods.

The dataset with optimal image quality was manually transferred to the DL model. The images of 3D maximal intensity projection, VR, CPR, and cross-sectional view of each major epicardial artery were first processed by AI software. The degree of stenosis along the longitudinal axis was determined by the diameter of the lumen in which the plaque was located and the diameter of adjacent normal vessels proximal and distal to the lesion. A cardiovascular radiologist (with 6 years of experience in cardiovascular imaging) independently recorded the DS values automatically quantified by an AI-aided model based on the 18-segment model of SCCT without knowing the clinical history and the results of ICA and manual CCTA quantification. The DS directly output from the DL-model was used for further comparison without manual adjustment.

### ICA and QCA analysis

ICA was performed with standard methods. At least two views were obtained for each major vessel. Two interventional cardiologists (with 10 and 15 years of experience in cardiovascular intervention), who were blinded to clinical histories and CCTA results, independently performed a visual assessment of DS based on the 18-segment model of SCCT in a vessel with a reference diameter ≥ 2.0 mm using two orthogonal views. The mean values of DS assessed by two interventional cardiologists were used for further analysis. QCA was performed independently by the same two readers as the visual assessment of ICA using a dedicated software (QAngio XA 2D, version 8.0.126.2; Medis Medical Imaging), with a 1-month washout period between visual and QCA assessments to avoid bias introduced by individual memory. Quantitative analysis was conducted on an 18-segment model of SCCT in vessels with a reference vessel diameter ≥ 2.0 mm, using the orthogonal view with optimal image quality for accurate lesion assessment. The software executed automated quantification of reference diameter and minimal lumen diameter. The DS was calculated by the software in accordance with the following formula: (1 − minimal lumen diameter/reference diameter) × 100. The mean values of DS measured by two interventional cardiologists were used for further analysis.

In patient-level, vessel-level, and segment-level analyses, the lesion exhibiting maximal DS within each respective category was selected when multiple lesions were present, with identical selection criteria applied to both CCTA and ICA.

### Statistical analysis

Statistical analysis was performed using MedCalc Statistical Software (version 20.019, MedCalc Software Ltd), IBM SPSS Statistics (version 26.0, IBM Corporation), and Python (version 3.9.7, Python Software Foundation). Continuous data were expressed as mean ± standard deviation (SD) or median and interquartile range (IQR), depending on whether they were normally distributed (tested with the Kolmogorov–Smirnov test). Categorical data were displayed as count (%). Agreement in DS quantification between the AI-assisted model, and expert interpretation of CCTA or visual assessment of ICA and QCA was assessed using the intraclass correlation coefficient (ICC) and Bland-Altman analysis. Weighted kappa was calculated to assess agreement for stenosis severity as categorical variables between the three methods and QCA. Correlation was assessed with Spearman’s correlation coefficient. Receiver operating characteristic (ROC) curve analyses were performed to calculate the area under the curve (AUC) of AI-based CT quantification, manual CT quantification, and visual assessment of ICA. The diagnostic performance of these quantitative methods for detecting obstructive stenosis was evaluated at two thresholds (DS ≥ 50% and ≥ 70%) against QCA using AUC and was compared with the DeLong method on a per-patient, per-vessel, and per-segment basis. Subgroup analyses were performed based on the coronary Agatston calcium score (CACS) (0, 1–99, 100–399, and ≥ 400). A two-tailed *p* < 0.05 was considered statistically significant.

## Results

### Clinical characteristics

**Fig. 1:**
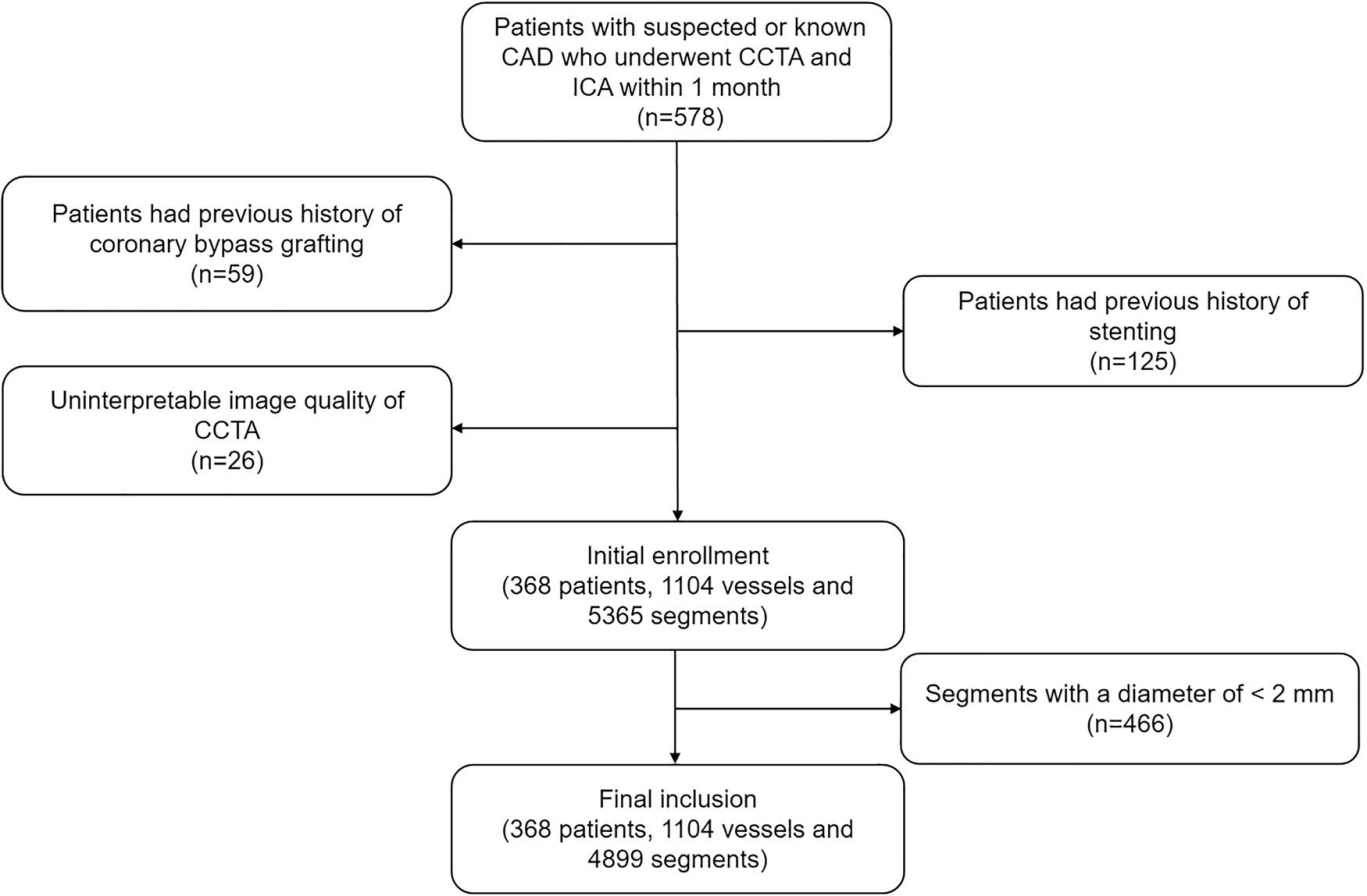
Flow chart of inclusion and exclusion. CAD, coronary artery disease; CCTA, coronary computed tomography angiography; ICA, invasive coronary angiography

A total of 578 patients with CCTA and ICA within 1 month were retrospectively reviewed. One hundred and eighty-four patients were excluded due to a previous history of coronary bypass grafting (59 cases) or stenting (125 cases). Twenty-six patients were excluded due to severely impaired image quality of CCTA. Furthermore, 1259 segments were anatomically absent, and an additional 466 segments measuring < 2 mm in diameter were excluded. Finally, a total of 368 patients (mean age 62.2 ± 10.0 years, range 30–88 years, 230 males) encompassing 1104 vessels and 4899 segments, were included in the analysis (Fig. 1). Detailed demographic data are presented in Table 1.

**Table 1:** Patient and scan characteristics

Parameters	
Ages (years)	62.2 ± 10.0
Male	230 (62.5%)
BMI (kg/m^2^)	24.8 ± 3.0
Risk factors	
Hypertension	239 (64.9%)
Diabetes mellitus	109 (29.6%)
Dyslipidemia	140 (38.0%)
Current smoking	81 (22.0%)
Agatston calcium score	
0	84 (22.8%)
1–99	110 (30.0%)
100–399	93 (25.3%)
≥ 400	81 (22.0%)
Disease pattern (≥ 50% CCTA DS)	
Single vessel	150 (40.8%)
Two vessels	104 (28.3%)
Three vessels	36 (9.8%)
CT scanners	
3rd generation DSCT	55 (14.9%)
2nd generation DSCT	108 (29.3%)
1st generation DSCT	30 (8.2%)
256-row WDCT	159 (43.2%)
320-row WDCT	16 (4.3%)

Values are mean ± SD or *n* (%)

Results are given according to patient-based analysis

*BMI* body mass index, *CCTA* coronary CT angiography, *DS* diameter stenosis, *DSCT* dual source CT, *SD* standard deviation, *WDCT* wide detector CT

### Diagnostic performance of AI-based CT quantification, manual CT quantification, and visual assessment of ICA

QCA revealed the presence of DS ≥ 50% in 79.1% (291 of 368) of patients, 40.9% (452 of 1104) of vessels, and 11.5% (564 of 4899) of segments. The DS ≥ 70% was found in 44.0% (162 of 368) of patients, 18.4% (203 of 1104) of vessels, and 4.4% (216 of 4899) of segments.

**Fig. 2:**
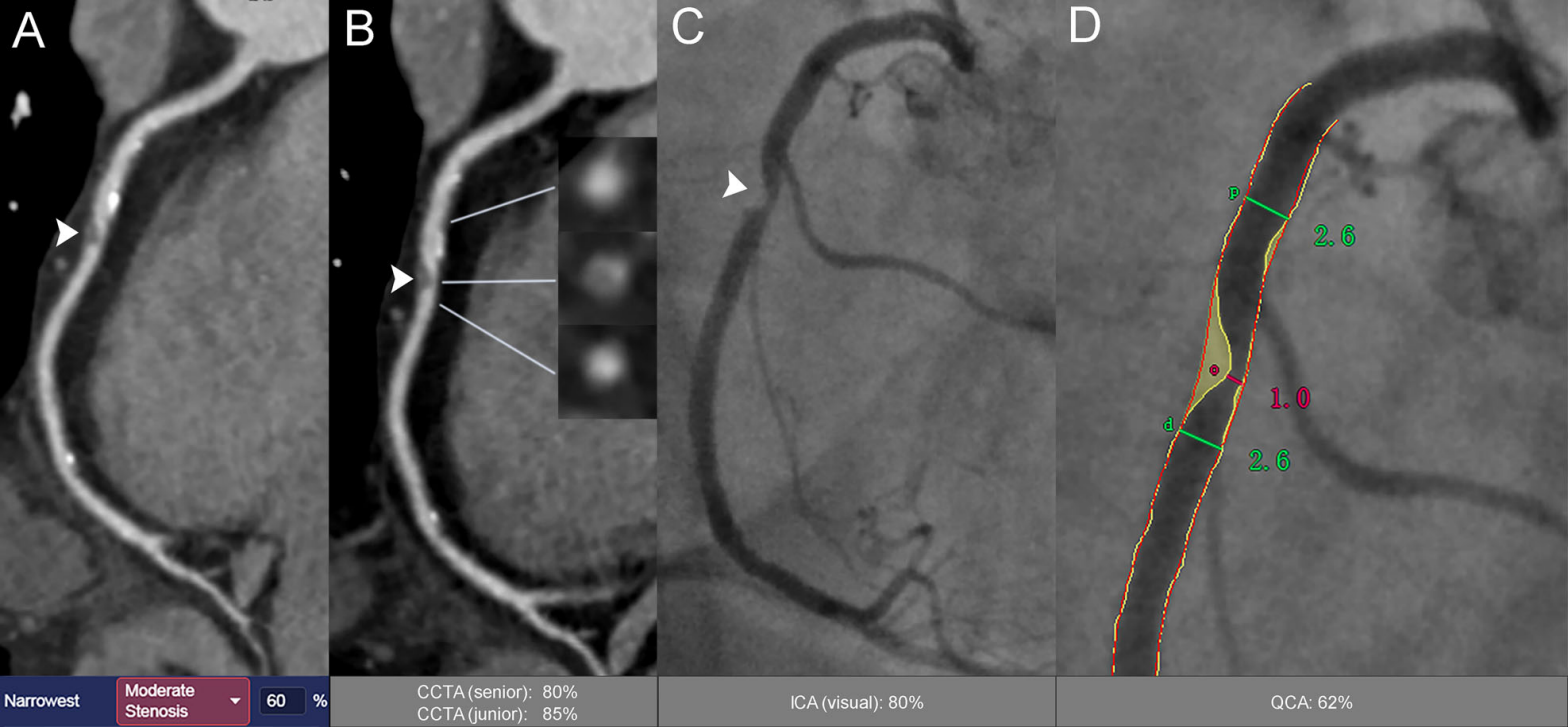
Representative case demonstrating concordance between AI-assisted CT stenosis quantification and QCA. **A**, **B** CCTA shows a mixed plaque at middle RCA (white arrowheads). **A** An AI-reconstructed CPR image. The bottom panel displays the result of AI-aided quantification for the lesion at the middle RCA (white arrowhead), indicating a 60% DS. **B** Manually reconstructed CPR image. The right panel shows cross-sectional views of RCA from top to bottom: proximal reference lumen, the narrowest luminal site, and distal reference lumen. The bottom panel displays manual CT quantification results: 80% DS by senior radiologists (CCTA (senior)) and 85% by junior radiologists (CCTA (junior)). **C** ICA shows the lesion at middle RCA (white arrowhead). The bottom panel gives the visual assessment (ICA (visual)) result of 80% DS. **D** QCA analysis of the same RCA lesion, measuring a proximal reference lumen diameter of 2.6 mm, a minimal lumen diameter of 1.0 mm, and a distal reference lumen diameter of 2.6 mm, yielding a DS of 62% (bottom panel). AI, artificial intelligence; CCTA, coronary computed tomography angiography; CPR, curved planar reformation; DS, diameter stenosis; ICA, invasive coronary angiography; QCA, quantitative coronary angiography; RCA, right coronary artery

**Fig. 3:**
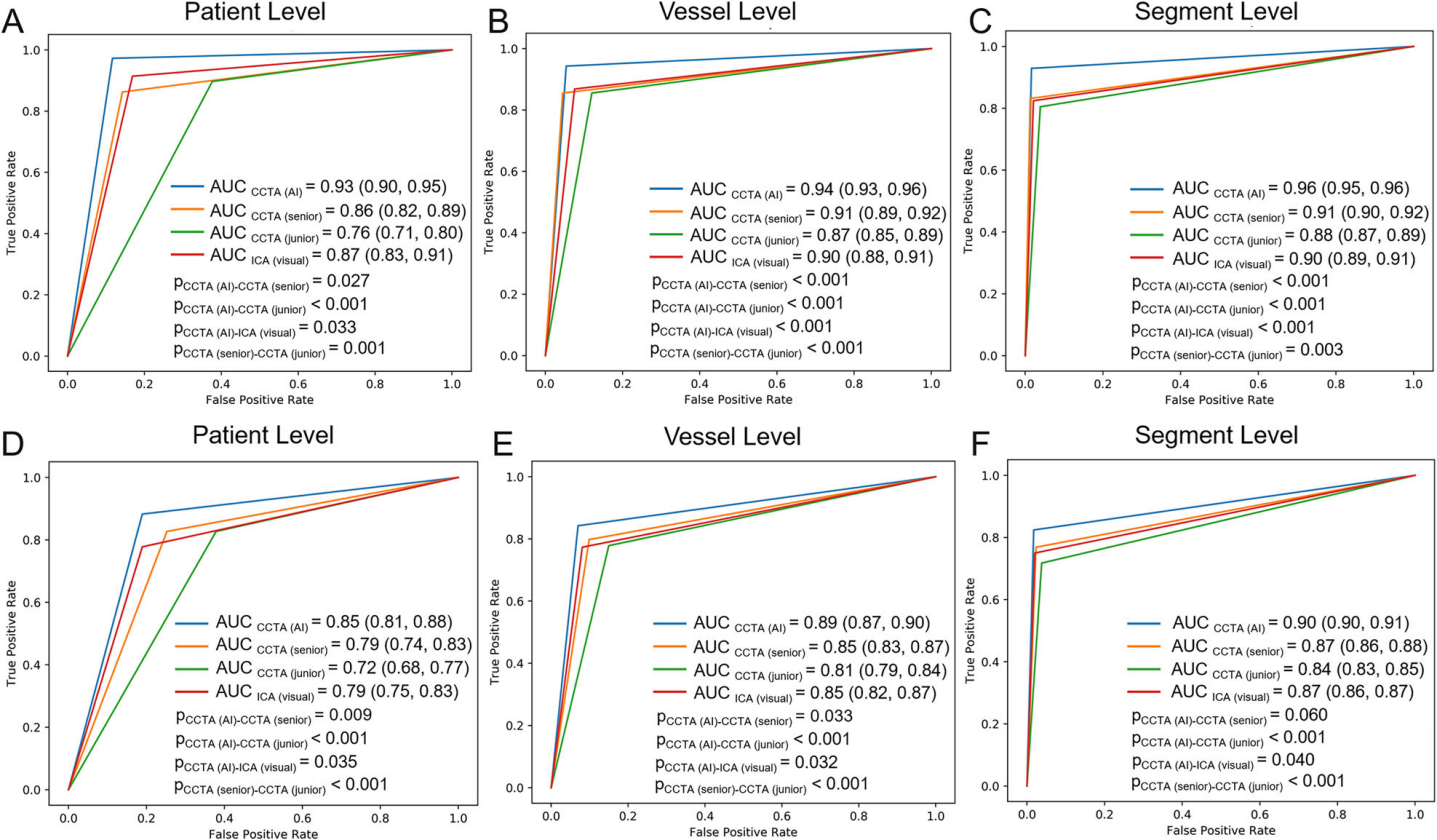
ROC analysis of different quantitative methods for detecting obstructive stenosis. **A**–**C** AUC values for the detection of DS ≥ 50% at the per-patient (**A**), per-vessel (**B**), and per-segment (**C**) levels, respectively. Comparisons include AI-based CT quantification (AUC_CCTA (AI)_), manual CT quantification by senior radiologists (AUC_CCTA (senior)_), manual CT quantification by junior radiologists (AUC_CCTA (junior)_), and visual assessment on ICA (AUC _ICA (visual)_). **D**–**F** AUC values for the detection of DS ≥ 70% at the per-patient (**D**), per-vessel (**E**), and per-segment (**F**) levels, respectively. For the detection of both DS ≥ 50% and ≥70%, the AUC differences between all pairwise comparisons were statistically significant, with the exception of the comparison between AUC_CCTA (AI)_ (0.90) and AUC_CCTA (senior)_ (0.87) at the segment level using the 70% threshold (*p* = 0.060). Values in parentheses represent 95% CIs. AI, artificial intelligence; AUC, area under the curve; CCTA, coronary computed tomography angiography; CI, confidence interval; DS, diameter stenosis; ICA, invasive coronary angiography; ROC, receiver operating characteristic

The AUC values of AI-based CT quantification were determined on a per-patient basis, per-vessel basis, and per-segment basis. The AUC values were 0.93, 0.94 and 0.96, respectively, for the detection of DS ≥ 50%, and 0.85, 0.89 and 0.90, respectively, for the detection of DS ≥ 70% (representative cases given in Fig. 2). For the detection of DS ≥ 50% and ≥ 70%, the AI-aided model significantly outperformed both manual CT quantification and visual assessment of ICA in terms of AUC at the patient, vessel, and segment levels (all *p* < 0.05), except for the comparison between the AI-aided model and manual CT quantification by senior radiologists at the segment level using the 70% threshold (AUC_CCTA (AI)_ : 0.90 vs AUC_CCTA (senior)_ : 0.87, *p* = 0.060) (Fig. 3). The accuracy, sensitivity, specificity, NPV, and PPV of AI-based CT quantification for identifying DS ≥ 50% on a per-patient level were 95.4%, 97.3%, 88.3%, 89.5% and 96.9%, respectively, and for identifying DS ≥ 70% were 84.2%, 88.3%, 81.1%, 89.8% and 78.6%, respectively (Tables 2 and 3).

**Table 2:** Diagnostic performance of CCTA-based DL, expert interpretation of CCTA, and visual assessment of ICA for detecting obstructive stenosis at 50% threshold

Levels	Methods	Accuracy (%) (95% CI)	Sensitivity (%) (95% CI)	Specificity (%) (95% CI)	NPV (%) (95% CI)	PPV (%) (95% CI)
Per-patient (*n* = 368)	CCTA _AI_	95.4 (92.7, 97.3)	97.3 (94.7, 98.8)	88.3 (79.0, 94.5)	89.5 (81.0, 94.4)	96.9 (94.4, 98.3)
	CCTA_senior_	86.1 (82.2, 89.5)	86.3 (81.8, 90.0)	85.7 (75.9, 92.6)	62.3 (55.0, 69.1)	95.8 (92.9, 97.5)
	CCTA_junior_	84.0 (79.8, 87.6)	89.7 (85.6, 92.9)	62.3 (50.6, 73.1)	61.5 (52.2, 70.1)	90.0 (87.1, 92.3)
	ICA_visual_	89.7 (86.1, 92.6)	91.4 (87.6, 94.4)	83.1 (72.9, 90.7)	71.9 (63.5, 79.1)	95.3 (92.6, 97.1)
Per-vessel (*n* = 1104)	CCTA_AI_	94.5 (93.0, 95.7)	94.3 (91.8, 96.2)	94.6 (92.6, 96.2)	95.9 (94.2, 97.2)	92.5 (89.9, 94.4)
	CCTA_senior_	91.4 (89.6, 93.0)	85.5 (82.0, 88.6)	95.5 (93.6, 97.0)	90.4 (88.2, 92.1)	93.1 (90.4, 95.1)
	CCTA_junior_	87.0 (84.8, 88.9)	85.5 (82.0, 88.6)	88.0 (85.2, 90.4)	89.6 (87.3, 91.5)	83.3 (80.2, 86.1)
	ICA_visual_	90.1 (88.2, 91.8)	86.8 (83.4, 89.8)	92.4 (90.1, 94.4)	90.9 (88.7, 92.7)	89.0 (86.0, 91.4)
Per-segment (*n* = 4899)	CCTA_AI_	97.8 (97.3, 98.2)	92.9 (90.5, 94.9)	98.4 (98.0, 98.8)	99.1 (98.8, 99.3)	88.4 (85.7, 90.6)
	CCTA_senior_	96.9 (96.4, 97.4)	83.2 (79.8, 86.2)	98.7 (98.4, 99.0)	97.8 (97.4, 98.2)	89.5 (86.7, 91.7)
	CCTA_junior_	94.3 (93.6, 95.0)	80.5 (77.0, 83.7)	96.1 (95.5, 96.7)	97.4 (97.0, 97.8)	73.0 (69.9, 75.9)
	ICA_visual_	96.1 (95.5, 96.6)	82.4 (79.1, 85.5)	97.9 (97.4, 98.3)	97.7 (97.3, 98.1)	83.6 (80.6, 86.3)

Methods include AI-based CT quantification (CCTA_AI_), manual CT quantification by senior radiologists (CCTA_senior_), manual CT quantification by junior radiologists (CCTA_junior_), and visual assessment on ICA (ICA_visual_)

*AI* artificial intelligence, *CCTA* coronary CT angiography, *ICA* invasive coronary angiography, *NPV* negative predictive value, *PPV* positive predictive value

**Table 3:** Diagnostic performance of CCTA-based DL, expert interpretation of CCTA, and visual assessment of ICA for detecting obstructive stenosis at 70% threshold

Levels	Methods	Accuracy (%) (95% CI)	Sensitivity (%) (95% CI)	Specificity (%) (95% CI)	NPV (%) (95% CI)	PPV (%) (95% CI)
Per-patient (*n* = 368)	CCTA_AI_	84.2 (80.1, 87.8)	88.3 (82.3, 92.8)	81.1 (75.0, 86.2)	89.8 (85.1, 93.1)	78.6 (73.3, 83.0)
	CCTA_senior_	78.3 (73.7, 82.4)	82.7 (76.0, 88.2)	74.8 (68.3, 80.5)	84.6 (79.6, 88.6)	72.0 (66.8, 76.7)
	CCTA_junior_	71.2 (66.3, 75.8)	82.7 (76.0, 88.2)	62.1 (55.1, 68.8)	82.1 (76.3, 86.7)	63.2 (58.7, 67.5)
	ICA_visual_	79.6 (75.1, 83.6)	77.8 (70.6, 83.9)	81.1 (75.0, 86.2)	82.3 (77.5, 86.2)	76.4 (70.6, 81.3)
Per-vessel (*n* = 1104)	CCTA_AI_	91.4 (89.6, 93.0)	84.2 (78.5, 89.0)	93.0 (91.1, 94.6)	96.3 (95.0, 97.3)	73.1 (68.0, 77.6)
	CCTA_senior_	88.2 (86.2, 90.1)	79.8 (73.6, 85.1)	90.1 (88.0, 92.0)	95.2 (93.8, 96.3)	64.5 (59.6, 69.2)
	CCTA_junior_	83.7 (81.4, 85.8)	77.8 (71.5, 83.3)	85.0 (82.5, 87.3)	94.5 (92.9, 95.7)	53.9 (49.6, 58.2)
	ICA_visual_	89.2 (87.2, 91.0)	77.3 (71.0, 82.9)	91.9 (89.9, 93.6)	94.7 (93.3, 95.9)	68.3 (63.0, 73.1)
Per-segment (*n* = 4899)	CCTA_AI_	97.6 (97.1, 98.0)	82.4 (76.7, 87.2)	98.3 (97.9, 98.6)	99.2 (98.9, 99.4)	68.7 (63.7, 73.3)
	CCTA_senior_	96.8 (96.2, 97.2)	76.9 (70.6, 82.3)	97.7 (97.2, 98.1)	98.9 (98.6, 99.2)	60.4 (55.5, 65.0)
	CCTA_junior_	95.1 (94.5, 95.7)	71.8 (65.3, 77.7)	96.2 (95.6, 96.7)	98.7 (98.4, 98.9)	46.7 (42.6, 50.9)
	ICA_visual_	97.0 (96.4, 97.4)	75.0 (68.7, 80.6)	98.0 (97.5, 98.4)	98.8 (98.5, 99.1)	63.0 (57.9, 67.9)

Methods include AI-based CT quantification (CCTA_AI_), manual CT quantification by senior radiologists (CCTA_senior_), manual CT quantification by junior radiologists (CCTA_junior_), and visual assessment on ICA (ICA_visual_)

*AI* artificial intelligence, *CCTA* coronary CT angiography, *ICA* invasive coronary angiography, *NPV* negative predictive value, *PPV* positive predictive value

There was discordance of > 30% between the AI-based CT quantification and QCA in 52 (4.0%) of 1284 segments with lesions revealed by QCA.

### Consistency analysis of AI-based CT quantification, manual CT quantification, and visual assessment of ICA compared with QCA

**Fig. 4:**
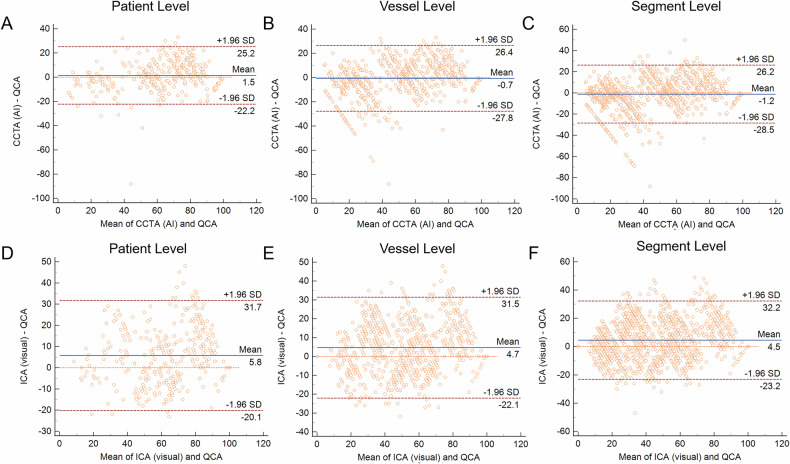
Bland-Altman analysis of agreement between different quantitative methods and QCA for DS measurement. **A**–**C** Agreement between AI-based CT quantification and QCA. Mean differences were 1.5% (95% CI: 0.2% to 2.7%), −0.7% (95% CI: −1.6% to 0.3%), and −1.2% (95% CI: −1.9% to −0.4%) at the per-patient (**A**), per-vessel (**B**), and per-segment (**C**) levels, respectively. **D**–**F** Agreement between visual assessment on ICA and QCA. Mean differences were 5.8% (95% CI: 4.4% to 7.2%), 4.7% (95% CI: 3.7% to 5.6%), and 4.5% (95% CI: 3.8% to 5.3%) at the per-patient (**D**), per-vessel (**E**), and per-segment (**F**) levels, respectively. AI, artificial intelligence; CCTA, coronary computed tomography angiography; DS, diameter stenosis; ICA, invasive coronary angiography; QCA, quantitative coronary angiography

There was a strong correlation between AI-based CT quantification of DS and QCA (*r* = 0.848 on a patient basis, *r* = 0.868 on a vessel basis, and *r* = 0.843 on a segment basis, all *p* < 0.001, Supplementary Figs. S1 and S2). Among these quantitative methods, AI-based CT quantification of DS demonstrated the highest agreement with QCA on a patient level (ICC = 0.861, *p* < 0.001), vessel level (ICC = 0.849, *p* < 0.001), and segment level (ICC = 0.845, *p* < 0.001, Supplementary Fig. S3). Bland-Altman analysis demonstrated that AI-based CT quantification of DS exhibited a mean difference of 1.5% (95% CI: 0.2% to 2.7%) per patient, −0.7% (95% CI: −1.6% to 0.3%) per vessel and −1.2% (95% CI: −1.9% to −0.4%) per segment compared with QCA, showing superior agreement over manual CT quantification and visual assessment of ICA (Fig. 4 and Supplementary Fig. S4). In the categorical agreement analysis of stenosis severity, AI-based CT quantification yielded a weighted kappa of 0.702 (per-patient), 0.699 (per-vessel), and 0.724 (per-segment) against QCA (Supplementary Fig. S3). Notably, all 25 segments classified as total occlusion by QCA were misdiagnosed as severe stenosis (DS ≥ 90%) by the AI-aided model.

### Subgroup analysis stratified by CACS categories

On a per-patient level, the AUC of CCTA-based quantification methods, particularly the AUC of manual quantification by junior radiologists, for detecting obstructive stenosis at both 50% and 70% thresholds showed a decreasing trend with increasing CACS, whereas visual assessment of ICA maintained stable AUC values across CACS strata. In the subgroup with CACS of 0, AI-based CT quantification demonstrated significantly higher AUC values than visual assessment on ICA at both the 50% and 70% stenosis thresholds (both *p* < 0.05) (Supplementary Figs. S5 and S6).

## Discussion

The study demonstrated that AI-based CT quantification achieved high diagnostic performance in detecting obstructive stenosis with good agreement against QCA, outperforming both manual CT quantification and visual assessment of ICA.

Previous multicenter studies have shown that, in comparison with QCA, CCTA exhibits high diagnostic accuracy in the evaluation of coronary artery stenosis [18, 19]. However, the diagnostic performance of CCTA for stenosis assessment was greatly affected by the interpretation experience of the readers [20, 21]. A multicenter study involving 4347 patients showed that experienced core laboratory readers identified 41% fewer patients with obstructive stenosis compared with less experienced side readers, and inconsistent interpretations were more prevalent with higher CACS [20]. In the present study, senior readers demonstrated significantly superior diagnostic performance to junior readers for identifying obstructive stenosis at both the 50% and 70% thresholds across all evaluated levels. This discrepancy in diagnostic accuracy for obstructive stenosis may be partially explained by the following factors. For borderline lesions, less experienced readers, due to lower confidence and heightened concern about missing significant stenosis, exhibit a tendency to over-diagnose these lesions as significant. Furthermore, in lesions with severe calcification, blooming artifacts on CCTA obscure the luminal contour, significantly complicating accurate stenosis assessment [22]. This effect is particularly challenging for less experienced readers, often leading to stenosis overestimation and increased inter-observer variability, consequently leading to increased rates of downstream testing.

DL utilizing convolutional neural networks (CNNs) demonstrates significant potential in cardiovascular imaging for tasks such as image segmentation and automated measurements of coronary arteries [11, 23]. Application of AI algorithms augments the quantity and robustness of information obtainable from cardiovascular images [23]. Emerging evidence highlights the pivotal role of AI-assisted algorithms in automated coronary artery stenosis detection and classification, improving the accuracy and efficiency of image interpretation [11]. Han et al observed that the diagnostic accuracy of AI-assisted algorithms in diagnosing obstructive stenosis was higher than that of an expert reader [24]. Similarly, another multicenter study showed that an AI-based algorithm outperformed an expert reader in detecting ≥ 50% stenosis at a per-vessel level [25]. Notably, these studies used visual assessment of ICA as the reference standard rather than QCA, which is more objective and reproducible. In the present study, AI-based CT quantification yielded AUCs of 0.93 and 0.85 for the detection of DS ≥ 50% and ≥ 70% on a per-patient basis with reference to QCA, outperforming manual CT quantification. The results align with and extend emerging evidence demonstrating the superiority of AI-enabled quantification over conventional methods in CCTA interpretation. The model employed in this study collectively enables comprehensive and reliable coronary image analysis: U-Net provides accurate lumen segmentation, V-Net preserves 3D spatial coherence for vessel reconstruction and stenosis localization, and the VGG architecture offers robust feature extraction for plaque detection. Due to the small calibers of coronary arteries, accurate assessment of stenosis on CCTA is challenging for human readers, particularly those with limited experience. DL-based models, developed from large databases of CT examinations and annotations, are capable of autonomously learning features and boundaries of segmentation targets such as vascular structures and plaques from CCTA images [26]. These models excel at capturing complex spatial hierarchies and subtle features within images—a task that poses significant challenges for human readers [11, 23]. Consequently, they allow accurate segmentation of coronary vessels, which serves as the foundation for precise quantitative stenosis analysis. The findings of our research suggest that the AI model has the capacity to support radiologists, particularly those with less experience, to achieve more accurate interpretations of CCTA in clinical practice.

At present, the most widely used approach for guiding revascularization is visual assessment of the degree of stenosis on ICA, which means that the accuracy of stenosis assessment is particularly important [2]. However, a previous study investigating the agreement between visual assessment of stenosis on ICA and QCA found that of 213 lesions considered ≥ 70% by visual assessment, 56 (26.3%) were < 70% by QCA. Mean difference in percent DS between visual assessment of ICA and QCA was 8.2 ± 8.4% [27]. Similarly, the mean difference between visual assessment and QCA was 5.8% on a per-patient basis in our study, indicating a positive bias. Another multicenter study conducted by Zhang et al showed that the discrepancies between visual assessment of ICA and QCA varied considerably at the hospital and physician level, indicating a high degree of heterogeneity in visual assessment [4]. In the present study, we observed that AI-based CT quantification exhibited high diagnostic performance for obstructive stenosis with good agreement against QCA, outperforming the visual assessment of ICA. The results indicated that AI-based CT quantification can assist in the visual assessment of stenosis in ICA. According to the guideline for coronary artery revascularization, visual estimation during ICA guides revascularization with a severity threshold of ≥ 70% (≥ 50% for left main) in patients with typical angina, whereas intermediate stenoses (40%–69%) require physiological assessment [28]. AI-based CT-derived fractional flow reserve (CT-FFR) has recently emerged as a noninvasive method for hemodynamic significance assessment of coronary lesions, providing information that complements anatomical stenosis severity and may improve overall evaluation of CAD [29]. Notably, visual assessment of ICA tends to overestimate stenosis severity, which may lead to unnecessary PCI procedures. In that case, AI-aided CCTA quantification is potentially useful to resolve the discordance between ICA and QCA, if pre-procedural CCTA data is available. When an AI-based CCTA strategy shows less tight stenoses than does visual analysis of ICA, further physiological evaluation (e.g., fractional flow reserve or AI-based CT-FFR) or QCA might be warranted to reduce unnecessary revascularization.

In view of the above findings, the clinical implications of the present study are as follows. Firstly, AI-aided CT quantification has a high diagnostic performance for diagnosing obstructive stenosis, suggesting that it can assist radiologists to make accurate interpretations of CCTA images in clinical practice. Nevertheless, it is imperative to scrutinize the diagnostic results of the AI-aided model to avoid underestimating lesions resulting in total occlusion. Furthermore, preoperative AI-aided CT quantification can be implemented as an adjunct to the visual assessment of ICA. In case of diagnostic discrepancy between AI-aided CT quantification and visual assessment of ICA concerning obstructive stenosis, further functional evaluation should be performed to guide the treatment strategy.

Despite the aforementioned advantages, it is worth mentioning that the AI model in our study was unable to accurately quantify segments with total occlusion. In the present study, all 25 segments that were classified as total occlusion by QCA were identified by the AI model as having DS ≥ 90%. To quantify stenosis, the AI model requires tracing a continuous vessel path along the coronary artery. In non-opacified occluded segments, the algorithm maintains this continuity by assuming a minimal residual lumen, which leads the lesion to be misinterpreted as near-complete rather than complete obstruction. Consequently, these lesions are classified as severe stenosis instead of total occlusion. In clinical practice, both categories represent high-risk obstructive CAD and should be referred for ICA and consideration of revascularization. Therefore, the stenosis downgrading of total occlusion by AI is unlikely to result in inappropriate clinical management.

Our study has several limitations. First, patients with a previous history of coronary bypass grafting or stenting were excluded, limiting the generalizability of our findings to this complex patient subgroup where artifact burden is often higher and the performance of the AI-based model may differ. Moreover, although we included data acquired by five models of CT scanners (3 models of dual-source CT and 2 models of wide detector CT), the images from mid-end CT scanners are not available in the current study. As high-end CT generates significantly better image quality than mid-end CT, it still needs further investigation on how this AI-based strategy may perform against QCA in a wider spectrum of CT scanners. In addition, the inclusion of patients who underwent both CCTA and ICA within one month may introduce selection bias at the patient level, potentially enriching the study population with individuals at a higher pre-test probability of CAD. This could affect the estimated diagnostic performance and limit the generalizability of our findings to broader clinical settings, particularly among lower-risk or screening populations. Nevertheless, the vessel- and segment-level analyses included a substantial proportion of non-obstructive lesions, which may partially mitigate this effect.

## Conclusions

AI-based CT quantification demonstrated excellent performance for diagnosing obstructive stenosis against QCA, outperforming manual CT quantification and visual assessment of ICA in most scenarios, with the exception of segment-level assessment of ≥ 70% DS compared with manual quantification by senior radiologists.

## Supplementary information

ELECTRONIC SUPPLEMENTARY MATERIAL

## Data Availability

The dataset used or analyzed during the current study are available from the corresponding author upon reasonable request.

## Abbreviations

AI: Artificial intelligence
CAD: Coronary artery disease
CCTA: Coronary CT angiography
DL: Deep learning
ICA: Invasive coronary angiography
PCI: Percutaneous coronary intervention
QCA: Quantitative coronary angiography
