# Supplementary material for: Coronary stenosis assessment: AI-based CT quantification, visual analysis of invasive angiography, and quantitative coronary angiography
Source: Insights Imaging. 2026 May 16;17:134. doi: 10.1186/s13244-026-02308-2 (PMC13180015; doi:10.1186/s13244-026-02308-2)
Supplement: Supplementary file 1 — ELECTRONIC SUPPLEMENTARY MATERIAL [file 13244_2026_2308_MOESM1_ESM.pdf]

Coronary stenosis assessment: AI-based CT quantification, visual analysis of invasive angiography, and quantitative coronary angiography

ELECTRONIC SUPPLEMENTARY MATERIAL

CCTA acquisition protocol

CCTA was performed on five CT scanners with 64-detector rows or greater from three vendors: (1) third-generation dual-source CT (SOMATOM Force, Siemens Heathineers); (2) second-generation dual source CT (SOMATOM Definition Flash, Siemens Heathineers); (3) first-generation dual source CT (SOMATOM Definition, Siemens Heathineers); (4) 256-row wide detector CT scanner (Revolution, GE Healthcare); (5) 320-row wide detector CT scanner (Aquilion ONE, Toshiba). The details of imaging parameters are listed below.

1) For third-generation dual-source CT (SOMATOM Force, Siemens Heathineers), prospective ECG-triggered acquisition was performed with the triggering window covering 35% to 75% of the R-R interval. Detector collimation was 96×0.6 mm. The gantry rotation time was 250 ms. Automated tube voltage and current modulation (CAREKv, CAREDose 4D, Siemens Healthineers) were applied. The reference tube current was set as 320 mAs and the reference tube

voltage was set as 100 kVp. Axial images were reconstructed with the smooth kernel (Bv 40) using the third-generation iterative reconstruction technique (strength 3, ADMIRE, Siemens Healthineers). The reconstructed slice thickness was 0.75 mm and reconstructed slice interval was 0.5 mm. The image phase with optimal image quality was subsequently selected and used for further analysis.

2) For second-generation dual-source CT (SOMATOM Definition Flash, Siemens Heathineers), prospective ECG-triggered acquisition was performed with the acquisition window covering 30% - 80% of the R–R interval. Detector collimation was 64 × 0.6 mm. The gantry rotation time was 280 ms. Tube voltage was 100 kVp for patients with body mass index (BMI) <30 kg/m², 120 kVp for patients with BMI ≥ 30 kg/m². Automated tube current modulation (CAREDose 4D, Siemens Healthineers) were applied. The reference tube current was set as 296 mAs. Axial images were reconstructed with the smooth convolution kernel (I26f) using the second-generation iterative reconstruction technique (strength 3, SAFIRE, Siemens Healthineers). The reconstructed slice thickness was 0.75 mm and reconstructed slice interval was 0.5 mm. The image phase with optimal image quality was subsequently selected and used for further analysis.

3) For first-generation dual-source CT (SOMATOM Definition, Siemens Healthineers), prospective ECG-gated acquisition was performed with an acquisition window covering 30%–80% of the R-R interval. Detector collimation was 32 × 0.6 mm. The gantry rotation time was 330 ms. Tube voltage was set at 120 kVp. Automated tube current modulation (CAREDose 4D, Siemens Healthineers) were applied. The reference tube current was set as 400 mAs. Axial images were reconstructed with a slice thickness of 0.75 mm and an interval of 0.5 mm using a medium-smooth convolution kernel (B26f). The image phase with optimal image quality was subsequently selected and used for further analysis.

4) For 256-row wide detector CT (Revolution CT, GE Healthcare), prospective ECG-triggered acquisition was performed. The triggering window was set at 35% to 75% of the R-R interval. The collimation was 256 × 0.625 mm. The gantry rotation time was 280 ms. Automated tube voltage selection (KV Assist, GE Healthcare) and tube current modulation (Smart mA, GE Healthcare) techniques were applied. Images were reconstructed using the standard algorithm with 40% of the adaptive statistical iterative reconstruction-v (ASIR-V, GE Healthcare) algorithm. The cardiac phase with the least coronary motion was selected, and the SSF motion correction algorithm was applied when necessary. The reconstructed slice thickness was 0.625 mm and reconstructed slice interval were 0.5 mm. The image phase with optimal image quality was

subsequently selected and used for further analysis.

5) For 320-row wide detector CT (Aquilion ONE, Toshiba Medical Systems), prospective ECG-triggered acquisition was performed with the triggering window covering 65% to 85% of the R-R interval. The collimation was 320 × 0.5 mm. The gantry rotation time was 350 ms. Tube voltage and tube current were determined using the automated exposure control function (Sure Exposure 3D, Toshiba Medical Systems). Depending on BMI, either 100 kVp or 120 kVp was used. Axial images were reconstructed with an FC03 algorithm using adaptive iterative dose reduction 3D (AIDR 3D, Toshiba Medical Systems). The reconstructed slice thickness was 0.5 mm and reconstructed slice interval was 0.25 mm. The image phase with optimal image quality was subsequently selected and used for further analysis.

AI-based CCTA quantification pipeline

Automated coronary stenosis quantification was performed using a commercially available, proprietary deep-learning-based platform whose architectures have been described in several previous studies [1, 2]. This deep-learning platform integrates multiple convolutional neural network architectures for coronary image analysis.

1) Cardiac and coronary segmentation

Cardiac chambers and coronary arteries were segmented using a modified volumetric V-Net architecture optimized for three-dimensional medical imaging. The model delineated major cardiac structures and extracted the coronary arterial tree, enabling automated classification of the three major coronary branches and 18 segments. The segmented cardiac volume was spatially normalized through cropping and resampling prior to further analysis. Coronary centerlines were subsequently extracted using a skeletonization algorithm, and anatomical labeling of coronary branches and segments was achieved by combining centerline topology with spatial relationships to segmented cardiac structures.

2) Plaque detection

To identify coronary plaque, cross-sectional image patches perpendicular to the coronary centerline were sampled at regular intervals to generate straightened vessel representations. Expert-annotated datasets were used to train a modified VGG-based deep-learning model that generated probability maps of plaque presence along the vessel axis. Calcified plaque was defined as lesions with attenuation ≥ 130 HU, whereas lesions with attenuation < 130 HU were classified as non-calcified; lesions containing both components were categorized as mixed plaques.

3) Lumen segmentation and stenosis quantification

Coronary lumen boundaries were segmented using a U-Net-based model trained on manually delineated cross-sectional images. Lumen diameters were estimated along the vessel centerline using multi-angle cross-sectional measurements to produce a continuous diameter profile. Diameter stenosis was quantified using a minimum-lumen-diameter-based formulation:

$$DS = 1 - 2 \times D_0 / (D_1 + D_2)$$

where D_0 denotes the minimal lumen diameter within the lesion and D_1 and D_2 represent reference diameters proximal and distal to the lesion, respectively. This definition is consistent with established angiographic quantification standards and ensures clinical interpretability.

These components constitute a fully automated pipeline enabling reproducible coronary segmentation, plaque detection, and stenosis measurement from CCTA datasets. These models were trained and validated prior to the present study on large annotated CCTA datasets, including multi-center data collected from 45 hospitals in China and comprising 9425 cases [2].

References

1 Zhang Y, Feng Y, Sun J et al (2024) Fully automated artificial intelligence-based coronary CT angiography image processing: efficiency, diagnostic capability, and risk stratification. Eur Radiol 34:4909-4919.

2 Xu L, He Y, Luo N et al (2021) Diagnostic Accuracy and Generalizability of a Deep Learning-Based Fully Automated Algorithm for Coronary Artery Stenosis Detection on CCTA: A Multi-Centre Registry Study. Front Cardiovasc Med. 8:707508.

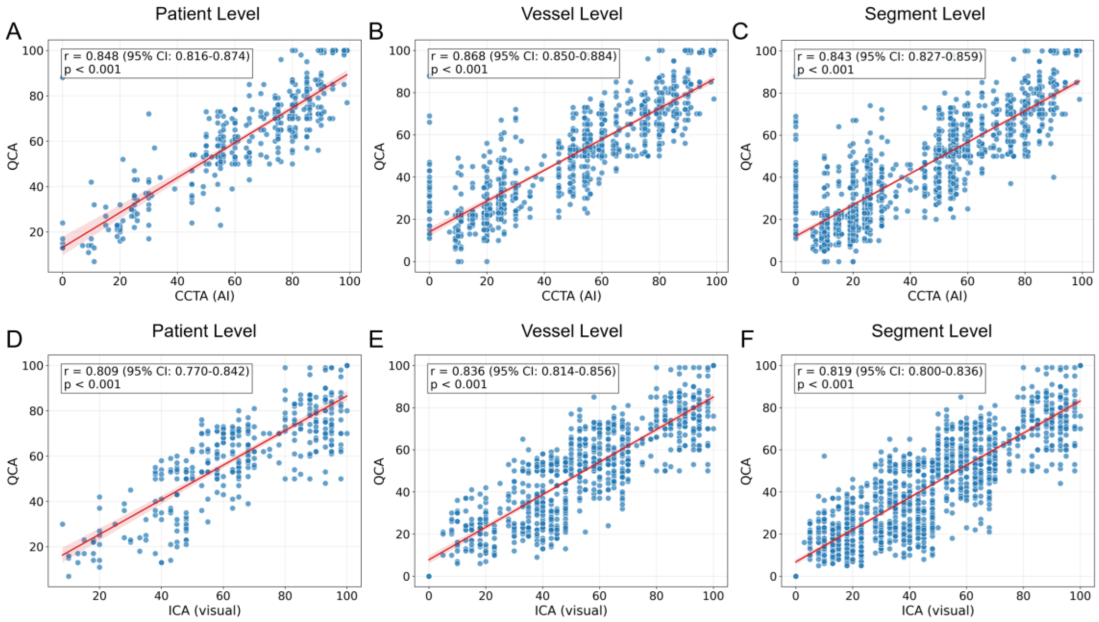

Supplementary Figure S1: Correlation of stenosis quantification by different methods with QCA for DS. (A-C) AI-based CT quantification vs. QCA at the per-patient (A), per-vessel (B), and per-segment (C) levels, respectively. (D-F) Visual assessment on ICA vs. QCA at the per-patient (D), per-vessel (E), and per-segment (F) levels, respectively.

Abbreviations: AI = artificial intelligence; CCTA = coronary CT angiography; DS = diameter stenosis; ICA = invasive coronary angiography; QCA = quantitative coronary angiography

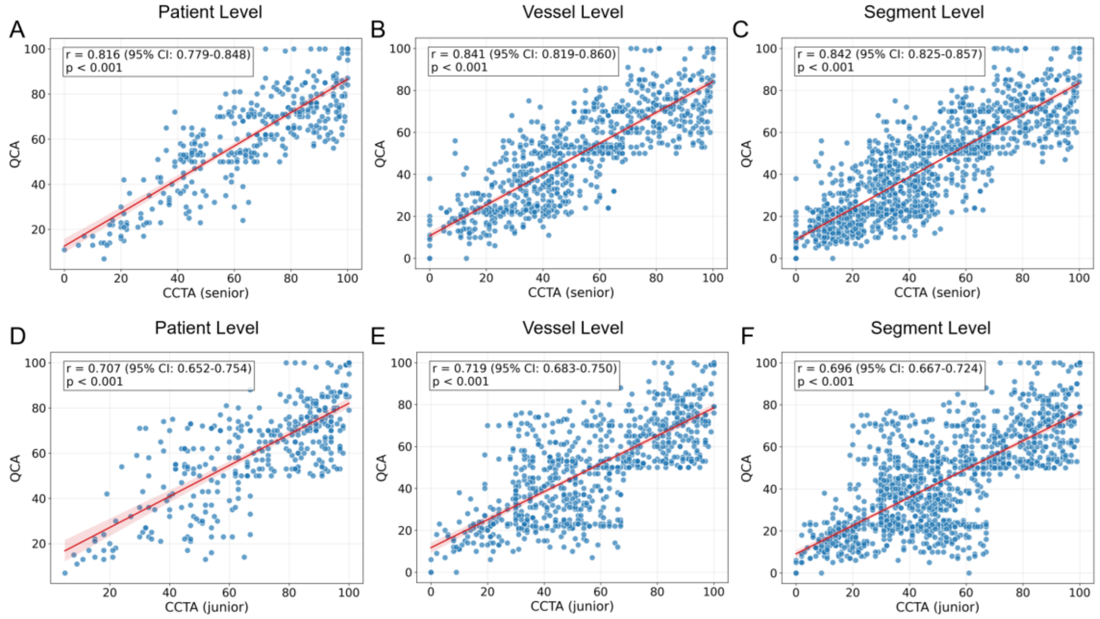

Supplementary Figure S2: Correlation of stenosis quantification by different methods with QCA for DS. (A-C) Manual CT quantification by senior radiologists vs. QCA at the per-patient (A), per-vessel (B), and per-segment (C) levels, respectively. (D-F) Manual CT quantification by junior radiologists vs. QCA at the per-patient (D), per-vessel (E), and per-segment (F) levels, respectively.

Abbreviations: AI = artificial intelligence; CCTA = coronary CT angiography; DS = diameter stenosis; ICA = invasive coronary angiography; QCA = quantitative coronary angiography

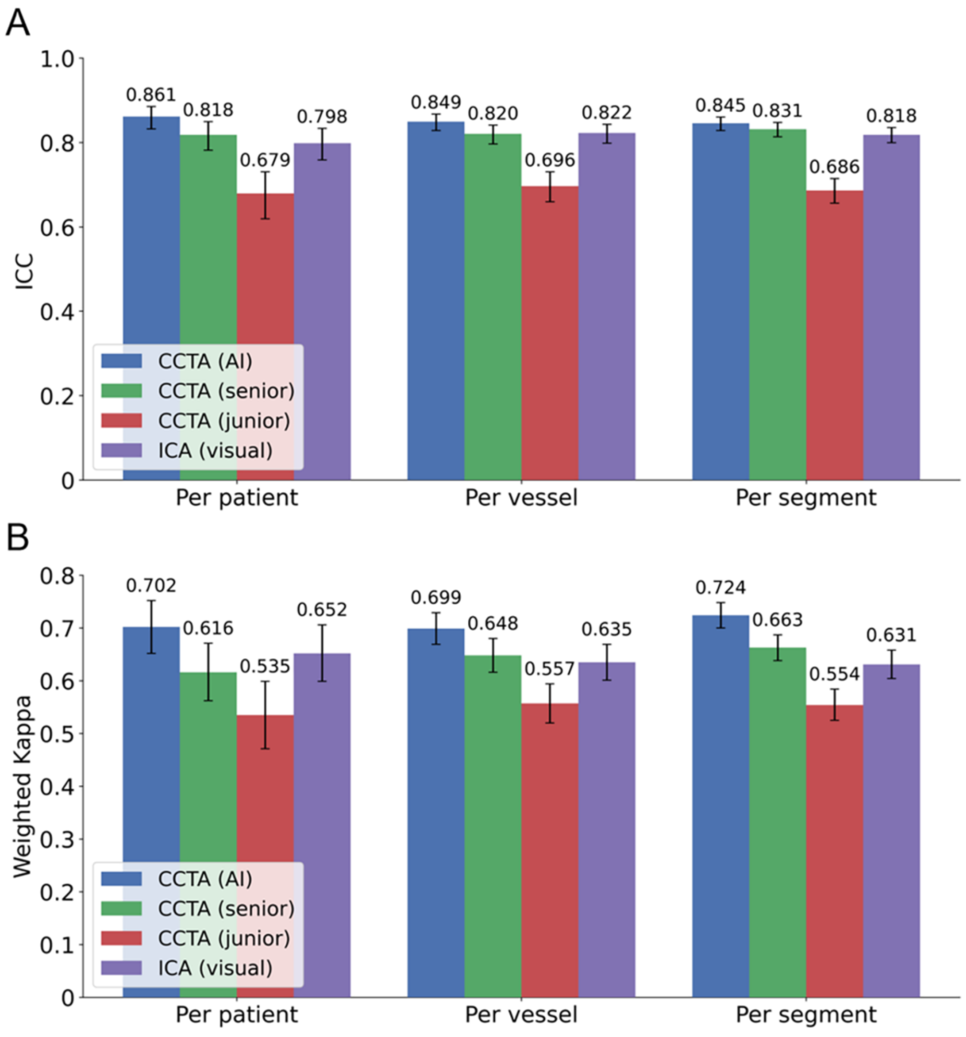

Supplementary Figure S3: Bar graphs show the agreement of stenosis assessment between different methods and QCA on different levels. (A) ICC for quantitative DS between QCA and each of the following: AI-based CT quantification (CCTA (AI)), manual CT quantification by senior radiologists (CCTA (senior)), manual CT quantification by junior radiologists (CCTA (junior)), or visual assessment on ICA (ICA (visual)) (all $p < 0.001$). CCTA (AI) demonstrated the highest ICC values at all levels. (B) Weighted kappa for stenosis category between QCA and each method. CCTA (AI) consistently

achieved the highest weighted kappa values across all levels. Error bars represent 95% CIs.

Abbreviations: AI = artificial intelligence; CCTA = coronary CT angiography; CI = confidence interval; DS = diameter stenosis; ICC = intraclass correlation coefficient; ICA = invasive coronary angiography; QCA = quantitative coronary angiography

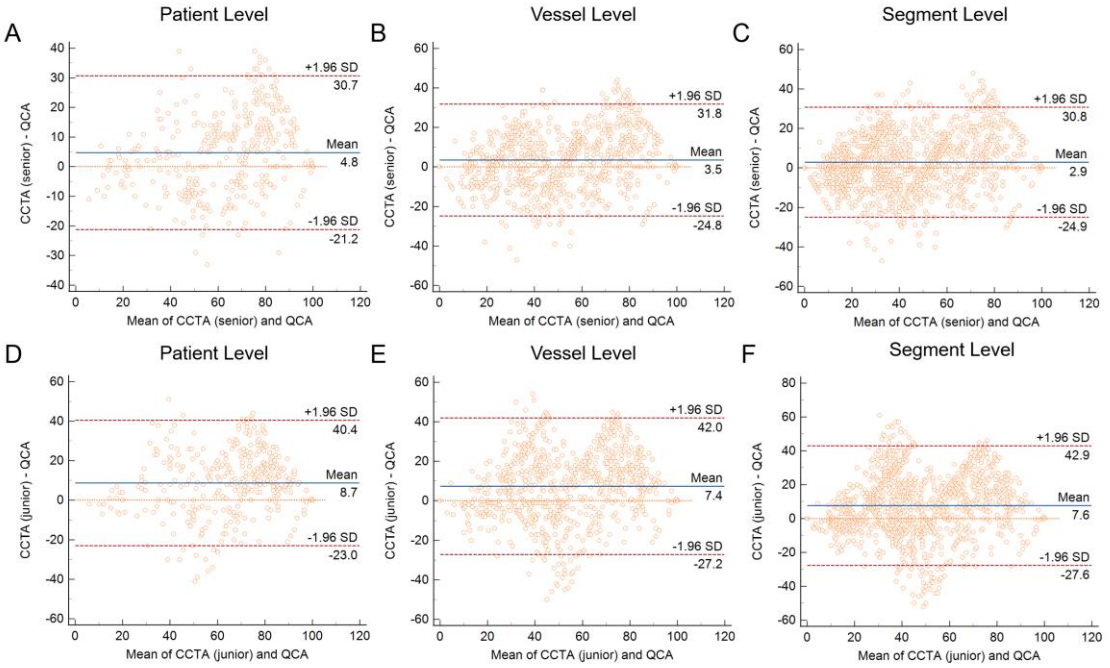

Supplementary Figure S4: Bland-Altman analysis of agreement between manual CT quantification and QCA for DS measurement. (A-C) Agreement between manual CT quantification by senior radiologists and QCA. Mean differences were 4.8% (95% CI: 3.4% to 6.1%), 3.5% (95% CI: 2.5% to 4.5%), and 2.9% (95% CI: 2.2% to 3.7%) at the per-patient (A), per-vessel (B), and per-segment (C) levels, respectively. (D-F) Agreement between manual CT quantification by junior radiologists and QCA. Mean differences were 8.7% (95% CI: 7.1% to 10.4%), 7.4% (95% CI: 6.2% to 8.6%), and 7.6% (95% CI: 6.6% to 8.6%) at the per-patient (D), per-vessel (E), and per-segment (F) levels, respectively.

Abbreviations: DS = diameter stenosis; QCA = quantitative coronary angiography; SD = standard deviation

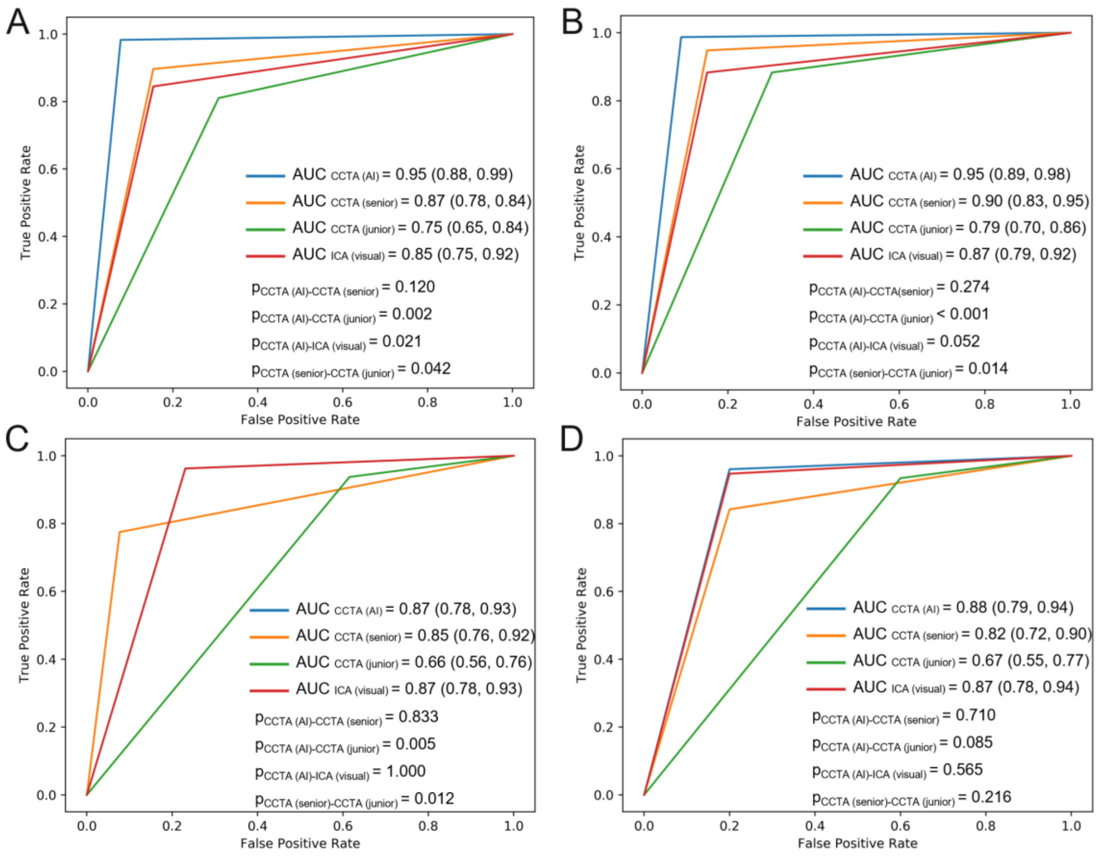

Supplementary Figure S5: ROC analysis of different quantitative methods for detecting obstructive stenosis at 50% threshold across CACS subgroups on a per-patient basis. (A-D) AUC values for the detection of DS ≥ 50% in subgroups with CACS of 0 (A), 1-99 (B), 100-399 (C), and ≥ 400 (D). Comparisons include AI-based CT quantification (AUC$_{\text{CCTA (AI)}}$), manual CT quantification by senior radiologists (AUC$_{\text{CCTA (senior)}}$), manual CT quantification by junior radiologists (AUC$_{\text{CCTA (junior)}}$), and visual assessment on ICA (AUC$_{\text{ICA (visual)}}$). In subgroups with CACS of 0, 1-99, and 100-399, both AUC$_{\text{CCTA (AI)}}$ and AUC$_{\text{CCTA (senior)}}$ were significantly greater than AUC$_{\text{CCTA (junior)}}$ (all $p < 0.05$). In the subgroup with CACS of 0, AUC$_{\text{CCTA (AI)}}$ was also significantly greater than AUC$_{\text{ICA (visual)}}$ ($p = 0.021$). There were no significant differences in the remaining pairwise comparisons (all $p > 0.05$). Values in parentheses represent 95% CIs.

Abbreviations: AI = artificial intelligence; AUC = area under the curve; CCTA = coronary CT angiography; CI = confidence interval; CACS = coronary Agatston calcium score; DS = diameter stenosis; ICA = invasive coronary angiography; ROC = receiver operating characteristic

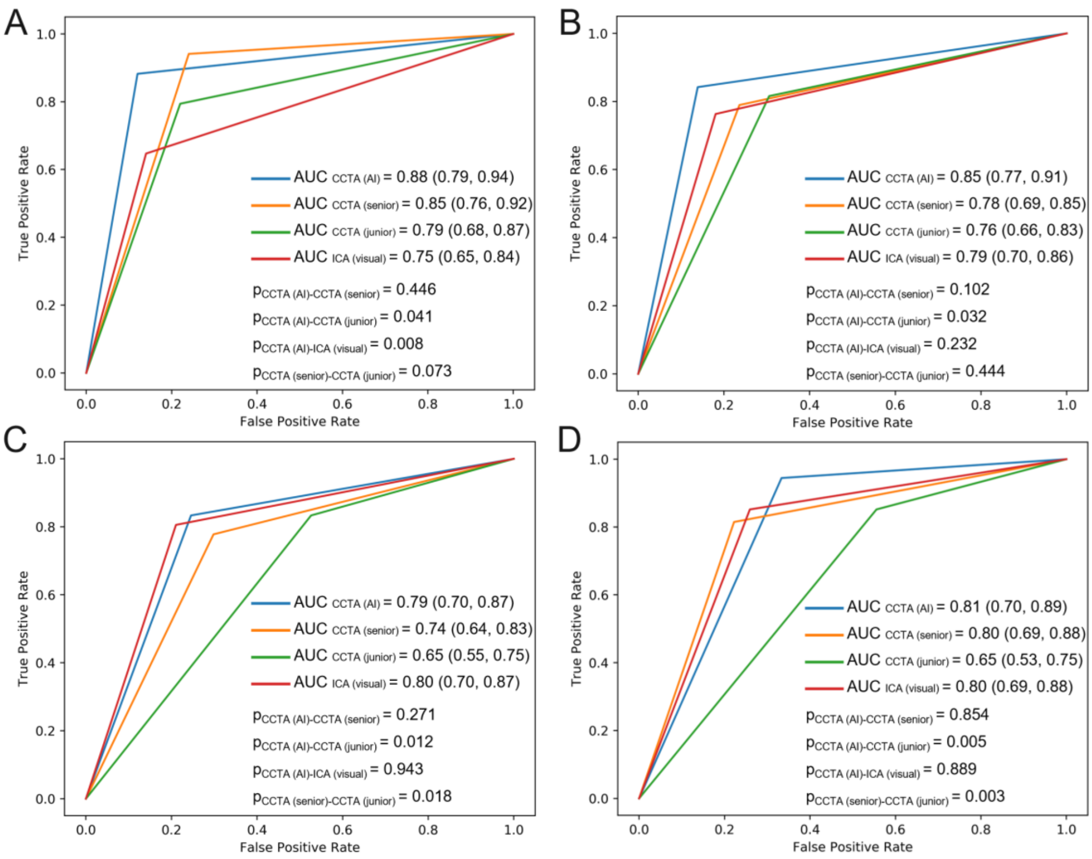

Supplementary Figure S6: ROC analysis of different quantitative methods for detecting obstructive stenosis at 70% threshold across CACS subgroups on a per-patient basis. (A-D) AUC values for the detection of DS ≥ 70% in subgroups with CACS of 0 (A), 1-99 (B), 100-399 (C), and ≥ 400 (D). Comparisons include AI-based CT quantification (AUC $_{\text{CCTA (AI)}}$), manual CT quantification by senior radiologists (AUC $_{\text{CCTA (senior)}}$), manual CT quantification by junior radiologists (AUC $_{\text{CCTA (junior)}}$), and visual assessment on ICA (AUC $_{\text{ICA (visual)}}$). AUC $_{\text{CCTA (AI)}}$ was significantly greater than AUC $_{\text{CCTA (junior)}}$ across all CACS subgroups (all $p < 0.05$). In subgroups with CACS of 100-399 and ≥ 400, AUC $_{\text{CCTA (senior)}}$ was significantly greater than AUC $_{\text{CCTA (junior)}}$ (both $p < 0.05$). In the subgroup with CACS of 0, AUC $_{\text{CCTA (AI)}}$ was significantly greater than AUC $_{\text{ICA (visual)}}$ ($p = 0.008$).

There were no significant differences in the remaining pairwise comparisons (all $p > 0.05$). Values in parentheses represent 95% CIs.

Abbreviations: AI = artificial intelligence; AUC = area under the curve; CCTA = coronary CT angiography; CI = confidence interval; CACS = coronary Agatston calcium score; DS = diameter stenosis; ICA = invasive coronary angiography; ROC = receiver operating characteristic
